# Imaging Flow Cytometry Protocols for Examining Phagocytosis of Microplastics and Bioparticles by Immune Cells of Aquatic Animals

**DOI:** 10.3389/fimmu.2020.00203

**Published:** 2020-02-18

**Authors:** Youngjin Park, Isabel S. Abihssira-García, Sebastian Thalmann, Geert F. Wiegertjes, Daniel R. Barreda, Pål A. Olsvik, Viswanath Kiron

**Affiliations:** ^1^Faculty of Biosciences and Aquaculture, Nord University, Bodø, Norway; ^2^Luminex B.V., ‘s-Hertogenbosch, Netherlands; ^3^Aquaculture and Fisheries Group, Wageningen University & Research, Wageningen, Netherlands; ^4^Department of Biological Sciences, University of Alberta, Edmonton, AB, Canada

**Keywords:** ImageStream^®X^, IFC, Atlantic salmon, Nile tilapia, blue mussel, phagocytosis

## Abstract

Imaging flow cytometry (IFC) is a powerful tool which combines flow cytometry with digital microscopy to generate quantitative high-throughput imaging data. Despite various advantages of IFC over standard flow cytometry, widespread adoption of this technology for studies in aquatic sciences is limited, probably due to the relatively high equipment cost, complexity of image analysis-based data interpretation and lack of core facilities with trained personnel. Here, we describe the application of IFC to examine phagocytosis of particles including microplastics by cells from aquatic animals. For this purpose, we studied (1) live/dead cell assays and identification of cell types, (2) phagocytosis of degradable and non-degradable particles by Atlantic salmon head kidney cells and (3) the effect of incubation temperature on phagocytosis of degradable particles in three aquatic animals–Atlantic salmon, Nile tilapia, and blue mussel. The usefulness of the developed method was assessed by evaluating the effect of incubation temperature on phagocytosis. Our studies demonstrate that IFC provides significant benefits over standard flow cytometry in phagocytosis measurement by allowing integration of morphometric parameters, especially while identifying cell populations and distinguishing between different types of fluorescent particles and detecting their localization.

## Introduction

Flow cytometry (FC) is widely employed for studying mammalian cells in particular and detecting biomarkers in clinical studies. FC systems quantify cell data within seconds and can provide information on cell phenotypes and functions. However, conventional FC is not designed to measure morphological and spatial information of single cells, and the technology is not able to efficiently detect dim and small particles (<300 nm) (1) as well as to distinguish aggregates of these small particles. Furthermore, although conventional FC can measure intra- and extra-cellular marker expressions, it does not provide information on marker localization. Another obstacle connected to conventional FC is auto-fluorescence. While the system cannot always precisely distinguish between false-positive and false-negative events (2), fine-tuning of instrument settings and protocol optimization can minimize the problem (3). Nevertheless, cell phenotype identification and functional analyses using conventional FC cannot be entirely objective as the equipment lacks image-capturing features. To overcome inaccuracies in acquiring cell data, quantitative studies preferably rely on both conventional FC and fluorescent cell imaging.

Imaging flow cytometry (IFC), also called multispectral imaging flow cytometry, is a powerful tool that enables us to collect information from single cells, including those from fluorescent images. Major advantages of IFC are (1) high fluorescence sensitivity, (2) high image resolution capability, (3) high speed processing, (4) ability to analyse changes in cell or nuclear morphology, (5) rare cell detection ability, and (6) capacity to understand cell-cell interaction (2). Certain disciplines of biology, namely hematology (4), immunology (5), cell biology (6), and microbiology (7) have already benefited from IFC. However, application of IFC is still in its infancy when it comes to studies in aquatic sciences.

Researchers have reported IFC-based analyses of fish cells, using nucleus staining to understand cell morphology and employing fluorescent particles to determine phagocytic activity in goldfish (8, 9). Different particles such as fluorescent latex beads (10), zymosan-APC (8), and nanoparticles (11) have been used to analyse phagocytosis using IFC. These methods can be further optimized, depending on the characteristics of the particles, e.g., latex beads that are not degraded vis-à-vis pHrodo™ BioParticles^®^ that emit fluorescent light upon acidification following ingestion by the target cells (10). Researchers have also improved the protocol for measuring particles' intensity in IFC (12). Overall, these studies provide the first information on the use of IFC to identify different cells and understand cell functions such as phagocytosis and the localization of markers of interest in cells from aquatic animals.

Though previous studies on aquatic animals have reported phagocytosis, here we present (1) basic, but optimized protocols for live/dead cell assay and identification of cell types (2), an improved protocol for examining phagocytosis of non-degradable (microplastic) and degradable (bioparticles) particles by immune cell types of fish, and (3) an optimized phagocytosis assay using cells harvested from three very different aquatic animals: cold water-adapted carnivorous marine fish (Atlantic salmon, *Salmo salar*), warm water-adapted omnivorous, freshwater fish (Nile tilapia, *Oreochromis niloticus*) and a cold water-adapted detritivorous/planktivorous marine mollusc (blue mussel, *Mytilus edulis*). Effect of incubation temperature was studied to verify the sensitivity and usefulness of the optimized phagocytosis assay.

## Methods

### Ethics Statement

The studies were approved (Atlantic salmon: FOTS ID 10050, Nile tilapia: FOTS ID 1042) by the National Animal Research Authority in Norway (Mattilsynet). The fish rearing and handling procedures were according to the approved protocols of FDU.

### Animals

Atlantic salmon (*S. salar*) in the weight range 700–900 g were used in this experiment. They were purchased from a commercial producer (Sundsfjord Smolt, Nygårdsjøen, Norway) and maintained at the Research Station of Nord University, Bodø, Norway. Fish were fed a commercial feed (Ewos AS, Bergen, Norway) and reared in a flow-through sea water system (temperature: 7–8°C, dissolved oxygen saturation: 87–92%, 24–h light cycle).

Nile tilapia (*O. niloticus*, 400–600 g) were bred and reared at the Research Station of Nord University in a freshwater recirculating aquaculture system (temperature: 28°C, pH: 7.6, dissolved oxygen saturation: 80% in outlet and 115% in inlet, 11 h dark/13 h light cycle). The fish were fed commercial feeds (Skretting, Stavanger, Norway) during the rearing period.

Adult blue mussels (*M. edulis*) were collected from a beach along the Saltenfjorden, Bodø, Norway (67°12′01″ N 14°37′56″ E) and transported to the Research Station, Nord University. Prior to isolation of hemocytes, they were kept for 2 days in running seawater at 7–8°C.

### Cell Isolation

Cells from salmon and tilapia head kidney (HK) were grown in Leibovitz's L-15 Medium (L-15; Sigma-Aldrich, Oslo, Norway), supplemented with 100 μg/mL gentamicin sulfate (Sigma), 2 mM L-glutamine (Sigma) and 15 mM HEPES (Sigma). Osmolality of medium was adjusted by adding a solution consisting of 5% (v/v) 0.41 M NaCl, 0.33 M NaHCO_3_, and 0.66 (w/v) D-glucose. Cell culture media were adjusted to 380 mOsm for salmon and 320 mOsm for tilapia. To culture the mussel hemocytes, filtered (through a 0.2 μm mesh) sea water was used as the medium.

Head kidney from salmon (*n* = 6) were sampled after the fish were killed with an overdose of MS-222 (Tricaine methane sulphonate; Argent Chemical Laboratories, Redmond, USA; 80 mg/L). Thereafter, the HK cells were isolated as described previously (13) with minor modifications. Briefly, HK was dissected out, and the tissues were transferred to 15 mL centrifuge tubes to make a total volume of 4 mL in ice-cold L-15+ (L-15 medium with 50 U/mL penicillin, 50 μg/mL streptomycin, 2% fetal bovine serum (FBS) and 10 U/mL heparin). The tissue was placed on a sterile 100 μM cell strainer (Falcon) and the cells were disrupted with the help of a syringe plunger. The harvested cells were washed twice in ice-cold L-15+. The cell suspension from salmon HK was then layered on 40/60% Percoll (Sigma) to separate HK leukocytes for magnetic-activated cell sorting (MACS) or layered on 34/51% Percoll to separate monocytes/macrophages for subsequent phagocytosis assays. After centrifugation (500 × g, 30 min, 4°C), the cells at the interface between the two Percoll gradients were collected and washed twice with ice-cold L-15-FBS free (L-15 medium with 50 U/mL penicillin, 50 μg/mL streptomycin) by centrifugation (500 × g, 5 min, 4°C). Cells were then kept in L-15+. HK phagocytic cells that were separated based on 34/51% Percoll gradient, were allowed to adhere on a petri dish for 3 days at 12°C. After removing the supernatant containing non-adherent cells, the petri dish with the adherent cells was placed on ice for 10 min, and the cells were collected by washing three times with 1.5 mL ice-cold PBS supplemented with 5 mM EDTA. Next, these collected cells were centrifuged (500 × g, 5 min, 4°C) and used for further analyses.

Head kidney from tilapia (*n* = 6) were collected after killing the fish with an overdose of clove oil (Sigma Aldrich, St. Louis, MO, USA), and cells were harvested as described previously (14, 15), with minor modifications. Briefly, the HK tissues were transferred to 15 mL centrifuge tubes to make a total volume of 4 mL in ice-cold L-15+. The cells were harvested from the HK and washed twice as described for salmon. The cell suspension was layered on 34/51% Percoll to separate phagocytic cells, and then after centrifugation, cells at the interface were collected and washed twice in L-15-FBS free. The cells in the suspension were allowed to adhere on a petri dish containing L-15+ for 3 days at 25°C. After removing the supernatant containing non-adherent cells, the petri dish with the adherent cells was placed on ice for 10 min, and the cells were collected by washing three times with 1.5 mL ice-cold PBS supplemented with 5 mM EDTA. Next, the collected cells were centrifuged (500 × g, 5 min, 4°C) and used for further analyses.

In the fish experiments, the cells were counted using a portable cell counter (Scepter™ 2.0 cell counter, EMD Millipore, Darmstadt, Germany).

Hemocytes from adult mussels (*n* = 6) were isolated as described previously (16) with minor modifications. Briefly, hemolymph was drawn from the posterior adductor muscle using a 2 mL syringe equipped with a 23G-needle. The hemocytes from each mussel were counted using a Neubauer chamber, and 0.2 × 10^6^ cells per sample were collected and re-suspended in 1 mL of filtered sea water to avoid formation of clumps.

### Magnetic-Activated Cell Sorting (MACS) of Salmon IgM^+^ Cells

The isolated salmon HK leukocytes (2 × 10^6^ cells) were incubated with mouse anti-trout/salmon IgM (6.06 μg/mL; Aquatic Diagnostics Ltd, Sterling, UK) for 60 min at 4°C. After two washes with L-15+, the cells were incubated for 15 min at 4°C in a cocktail with a total volume of 100 μL, which contained L-15+, 1 μL of goat anti-mouse IgG-FITC (0.75 mg/mL; Thermo Fisher Scientific, Oslo, Norway) and 40 μL of goat anti-mouse IgG microbeads as per the instructions of the manufacturer (Miltenyi Biotec, Bergisch Gladbach, Germany). First, MACS LD columns (Miltenyi Biotec) that were placed in a magnetic separator of the multistand were washed using L-15+. The cell suspension was then transferred into the LD column. Following cell sorting, the positive cells were harvested and re-suspended in L-15+.

### Live/Dead Cell Assay

In the studies on cells from salmon and tilapia, aliquots containing 1 × 10^6^ cells in 50 μL PBS were transferred to 1.5 mL microcentrifuge tubes. Then 1 μL of propidium iodide (PI; 1 mg/mL, Sigma) was added to each sample to detect the dead cells in the cell suspension. In the case of mussel, aliquots containing 0.2 × 10^6^ cells in 50 μL filtered sea water were transferred to 1.5 mL microcentrifuge tubes, and then 1 μL of DRAQ5™ (25 mM, Thermo Fisher Scientific) was added to each sample to detect the dead cells in the cell suspension. The tubes were gently mixed before the samples were run through the ImageStream^®^^X^ Mk II Imaging Flow Cytometer (Luminex Corporation, Austin, TX, USA). Cell analyses were performed on 10,000 cells acquired at a rate of 300 objects/second at low speed and a magnification of 40×. Dead cells were estimated as the percent of cells positive for either PI or DRAQ5™ (red fluorescent cells). After excluding the dead cells, viable cells were analyzed to generate brightfield (BF) area (size) vs. side scatter (SSC) intensity (complexity) dot plots. Instrument settings were kept identical throughout the study.

### Phagocytosis Assay

In the first phagocytosis experiment, phagocytic cells from salmon HK were employed to study the uptake of two types of particles; non-degradable fluorescent polystyrene microplastic beads (2.1 μm; Magsphere Inc., California, USA) and degradable fluorescent bio-particles (>0.2 μm; pHrodo™ Red *Escherichia coli* Bioparticles, Thermo Fisher Scientific). In the second experiment, we used degradable fluorescent bio-particles only; to determine phagocytic ability and capacity of the cells from salmon, tilapia and mussel at two different incubation temperatures. Phagocytic ability was measured as the percent of phagocytic cells among the total macrophage-like cells or hemocytes. On the other hand, phagocytic capacity was measured as the mean number of particles per phagocytic cell. Phagocytic index (PI) or phagocytic activity was determined employing the equation (17, 18):

Phagocytic index (PI) = [% phagocytic cells containing at least one particle] × [mean particle count per phagocytic cell].

Briefly, fluorescent bio-particles were added at a cell:particle ratio of 1:5 per sample, both in the case of HK macrophages (0.5 × 10^6^ cells) and hemocytes (0.2 × 10^6^ cells). Cells suspensions and bio-particles were mixed and incubated for 2 h at different temperatures. Following incubation, cell suspensions were washed twice with 500 μL L-15+ by centrifugation (500 × g, 5 min, 4°C). The supernatant was discarded, and the resulting cell pellets were re-suspended in 50 μL PBS. The cell samples were run in an imaging flow cytometer (Luminex), equipped with a 10 mW 488 nm argon-ion laser, to detect the bio-particle fluorescence (577/35 nm bandpass; Channel 3). Thereafter, the images were analyzed using IDEAS 6.1.822.0 software (Luminex).

### Data and Statistical Analyses

Statistical analysis was performed in RStudio version 1.1.463. Normality of the data was tested by Shapiro-Wilk Test, and the assumption of equal variance was checked by Bartlett's Test. Comparisons between the two groups were performed using unpaired Student's *t*-test. Statistically significant differences (*p* < 0.05) are reported for the phagocytosis data.

## Results

### Live/Dead Cells and Leukocyte Populations From Salmon Head Kidney

**Figure 1:**
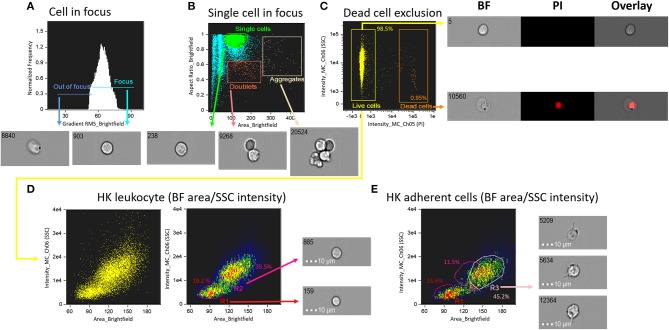
Schematic of the gating strategy to determine areas and populations of head kidney cells from Atlantic salmon. **(A)** Focused cells were gated using IDEAS^®^ gradient RMS feature which helps to select high-quality images with RMS values above 50. **(B)** Representative image showing the gated cells; single cells, doublets and aggregates are shown in a dot plot. Aspect ratio is a feature that evaluates an elliptical shape fitted around the detected object. The ratio is calculated as the minor axis divided by the major axis; a value close to 1 indicates that the cell has a circular shape, while a value in the range 0.4–0.8 signals the presence of doublets or aggregates if the individual cells are of oval morphology. **(C)** Orange dots are the dead cells that were excluded using propidium iodide, to separate the live cells (yellow dots). **(D)** HK leukocyte population is shown in a brightfield (BF) area (cell size) vs. side scatter (SSC) intensity (cell internal complexity) plot. **(E)** HK adherent cell (macrophage-like cell) population is shown in a BF area vs. SSC intensity plot. All cell images were captured with 40 × objective. Scale bar = 10 μm. BF, brightfield; PI, propidium iodide; R, region; R1, lymphocyte-like cells; R2, monocytes/macrophages-like cells; R3, macrophage-like cells.

To determine single cell area and to identify cell populations, we employed a basic gating strategy using the Brightfield Gradient Root Mean Square (RMS) feature of the imaging flow cytometer (see Figure 1). This strategy helped us to select the cells in best focus, i.e., this allowed us to obtain high quality images with RMS values >50 (Figure 1A). Next, we separated single cells from others (debris, doublets and aggregates; Figure 1B). Dead cells were excluded based on positivity for PI (Figure 1C). The percentage of live cells were 98.5%. The brightfield (BF) area and side scatter (SSC) intensity of the live, single cells were assessed. We prepared a BF area vs. SSC intensity dot plot to show the salmon HK leukocyte populations (Figure 1D). Cells with smaller size (low BF area) and low SSC intensity were possibly lymphocyte-like cells (19.2%; R1 in Figure 1D) while those with larger size (BF area) and higher SSC intensity compared to lymphocyte-like cells were considered as monocytes/macrophages (35.5%; R2 in Figure 1D). Figure 1E shows salmon HK adherent cell populations in a BF area vs. SSC intensity dot plot; here, R3 is probably HK macrophage-like cells (45.2%). We conclude that using IFC, dead cells can be excluded, and different single cell populations can be better detected than in conventional FC.

### Salmon Head Kidney IgM^+^ Lymphocyte Identification

**Figure 2:**
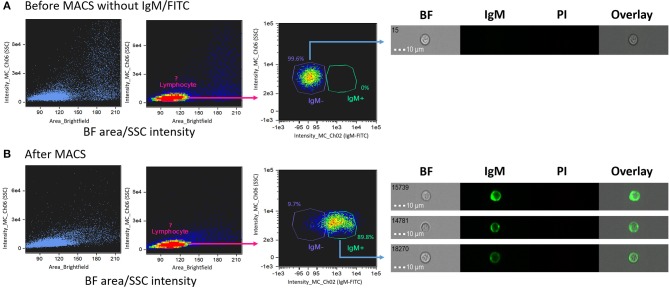
Identification of salmon IgM^+^ lymphocyte after magnetic-activated cell sorting (MACS). Dot plots of **(A)** HK leukocytes before and **(B)** after MACS separation. The left and middle plots in **(A,B)** show brightfield (BF) area vs. SSC intensity and the right plot in **(A,B)** shows the density of the target cells. Cells without IgM-FITC are shown in the IgM^−^ gate (**A**, right panel) while IgM^+^ cells after MACS are shown in the IgM^+^ gate (**B**, right panel). All cell images were captured with 40 × objective. Scale bar = 10 μm. BF, brightfield; IgM, immunoglobulin M; PI, propidium iodide.

Salmon head kidney IgM^+^ lymphocytes separated using MACS were used to ascertain their localization in a BF area vs. SSC intensity dot plot (Figure 2). For this purpose, cells were extracellularly stained with IgM-FITC, which enabled us to identify areas of negatively- and positively-stained B lymphocyte populations. Before MACS (Figure 2A), all cells were located in the IgM^−^ area (right panel Figure 2A). After staining with IgM-FITC and performing MACS (Figure 2B), most cells were located in the IgM^+^ area (89.8%; right panel Figure 2B). These data confirmed that the IgM^+^ cells matched the location of the lymphocyte-like cells (R1 population in Figure 1D). Thus, we confirmed the localization of salmon IgM^+^ cells using IFC.

### Examining Phagocytosis Using Non-degradable Fluorescent Microplastic Beads

**Figure 3:**
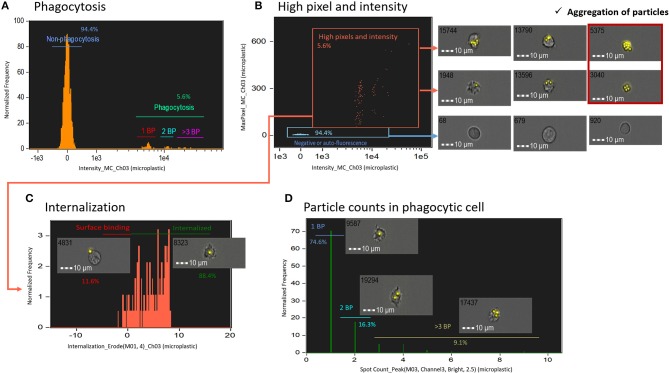
Measurement of phagocytosis using non-degradable fluorescent particles (microplastics). **(A)** A histogram of fluorescent microplastic intensity in live cells. **(B)** To exclude auto-fluorescence and find the best images, images with more pixels and high intensity were gated. Errors may occur in conventional FC due to aggregation of microplastics. **(C)** Internalized cells were gated using internalization score. **(D)** Cells with internalized particles were plotted in a histogram of spot count, which shows the number of particles. All cell images were captured with 40 × objective. Scale bar = 10 μm. BP, bio-particle.

To determine the phagocytosis of microplastics by salmon HK cells, first, we plotted histograms of fluorescence intensity of non-degradable fluorescent polystyrene microplastic beads in live cells (Figure 3A). Because all the polystyrene beads were of similar size, we assumed that fluorescence intensity is proportional to the number of beads taken up by each phagocytic cell. Using IFC, we could exclude auto-fluorescence and could gate images with more pixels and higher intensity (phagocytic images with pixel value > 30 were considered to be of high quality) (Figure 3B). Caution was taken to exclude aggregates; addition of many microplastic beads can cause bead aggregation, leading to false identification of aggregates as phagocytic cells, especially in conventional FC. Next, we gated phagocytic cells that engulfed microplastic beads using an internalization score (Figure 3C). This score is the ratio of the particle intensity inside a cell to the intensity of the whole cell, and it is calculated after masking (which selects pixels within an image based on their intensity and localization) with the following mask function [Erode (M01, 4)_Ch03]. The ratio indicated the proximity of microplastic to the center of the cell; cells with a score of > 0.3 were considered to have internalized particles and those with a score of < 0.3 were considered to have surface-bound particles (11). Finally, only cells with internalized particles were presented in a histogram of spot count feature, which is an ideal approach to quantify the masked spots in the cell (Figure 3D). Overall, IFC can be applied for detecting non-degradable microplastic beads inside the phagocytic cells and quantifying the number of beads. In addition, salmon HK phagocytic cells could recognize microplastics as foreign bodies although we observed only few phagocytosed particles.

### Examining Phagocytosis Using Degradable Fluorescent Bio-particles

**Figure 4:**
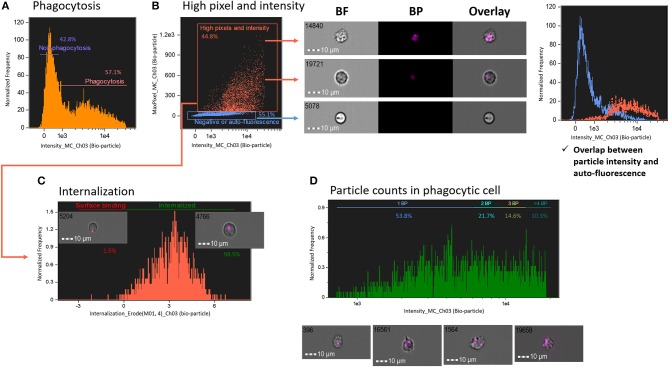
Measurement of phagocytosis using degradable fluorescent particles (pHrodo™). **(A)** Live cells are plotted in a histogram of fluorescent bio-particle intensity. **(B)** To exclude auto-fluorescence and find the best image, images with more pixels and high intensity were gated. The histogram (right panel) points to the detection error caused by overlapping curves of particle intensity (orange) and auto-fluorescence (blue) in conventional flow cytometry. **(C)** Internalized cells were gated using internalization score. **(D)** Cells with internalized particles were plotted in a histogram of bio-particle intensity, which shows the number of particles. All cell images were captured with 40 × objective. Scale bar = 10 μm. BF, brightfield; BP, bio-particle.

To determine phagocytosis of degradable bio-particles by salmon HK cells compared with non-degradable microplastics, first, we plotted histograms of fluorescence intensity of degradable bio-particles (Figure 4A). In comparison to the histogram of the non-degradable microplastic beads described above (Figure 3), it was more difficult to distinguish the number of bio-particles in this histogram. To exclude auto-fluorescence and obtain high-quality images, we adopted a gating strategy based on high pixel (pixel value > 30) and intensity of images (Figure 4B). We created two gates, one to include particles with high pixel and high intensity and the other one with negative or auto-fluorescence (histogram in Figure 4B). From the histogram, it is clear that overlapping particle intensity (orange) and auto-fluorescence (blue) curves can cause detection errors. Cells that had engulfed the bio-particles were gated using the internalization score as described in the previous section (Figure 4C). Finally, only cells with internalized particles are presented in a histogram of particle intensity to understand the number of particles in the phagocytic cells (Figure 4D). We found that to quantify the number of degradable particles, particle intensity-based protocol is a better strategy compared to the method employing spot count feature.

### Optimizing IFC-Based Method for Phagocytosis Assay

**Figure 5:**
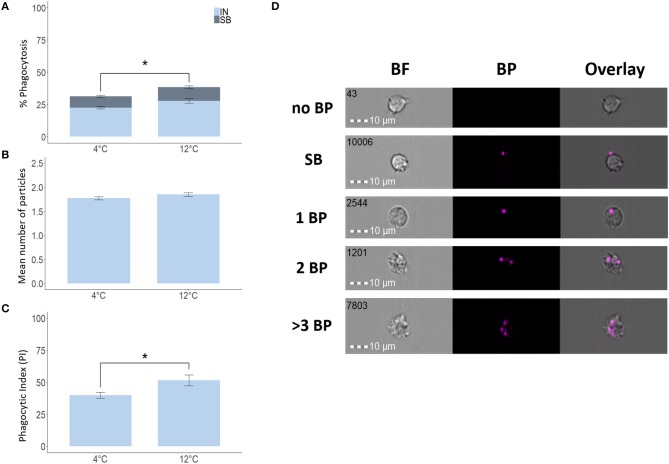
Phagocytosis of Atlantic salmon head kidney macrophages incubated at different temperatures. Percent of phagocytic cells **(A)**, mean number of particles ingested per phagocytic cell **(B)** and phagocytic index, PI **(C)**. Representative cell images that show cells with no BP, SB, and 1BP, 2BP, and > 3BP **(D)**. Statistically significant differences (*p* < 0.05) are indicated using asterisks. Bar plots show the mean ± SD. Sample size = 6. All cell images were captured with 40 × objective. Scale bar = 10 μm. IN, internalization; SB, surface-binding particles; 1 BP, 2 BP, and > 3 BP, 1–3 internalized bio-particles; BF, brightfield.

**Figure 6:**
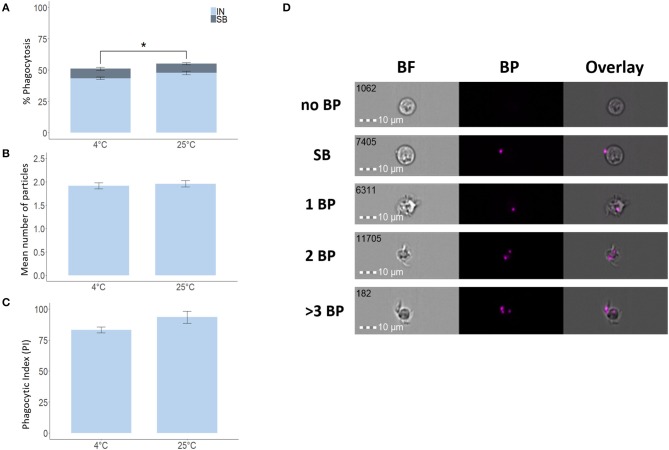
Phagocytosis of Nile tilapia head kidney macrophages incubated at different temperatures. Percent of phagocytic cells **(A)**, mean number of particles ingested per phagocytic cell **(B)** and phagocytic index, PI **(C)**. Representative cell images that show cells with no BP, SB, and 1BP, 2BP, and > 3BP **(D)**. Statistically significant differences (*p* < 0.05) are indicated using asterisks. Bar plots show the mean ± SD. Sample size = 6. All cell images were captured with 40 × objective. Scale bar = 10 μm. IN, internalization; SB, surface-binding particles; 1 BP, 2 BP, and > 3 BP, 1–3 internalized bio-particles; BF, brightfield.

**Figure 7:**
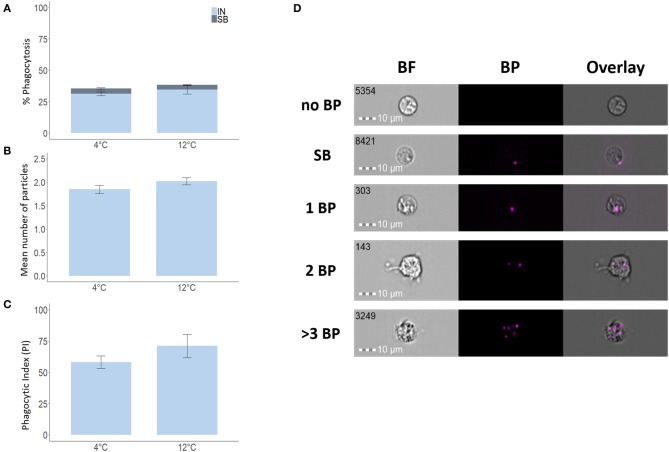
Phagocytosis of blue mussel hemocytes incubated at different temperatures. Percent of phagocytic cells **(A)**, mean of number of particles ingested per phagocytic cell **(B)**, and phagocytic index, PI **(C)**. Representative cell images that show cells with no BP, SB, and 1BP, 2BP, and > 3BP **(D)**. Statistically significant differences (*p* < 0.05) are indicated using asterisks. Bar plots show the mean ± SD. Sample size = 6. All cell images were captured with 40 × objective. Scale bar = 10 μm. IN, internalization; SB, surface-binding particles; 1 BP, 2 BP, and > 3 BP, 1–3 internalized bio-particles; BF, brightfield.

To verify the validity of our IFC-based method, we used degradable fluorescent bio-particles from *E. coli* to assess the effect of incubation temperature on the phagocytic activity and capacity of phagocytic cells from three aquatic animals. The phagocytic ability of HK phagocytic cells from salmon (Figure 5A) and tilapia (Figure 6A) incubated at 12 and 25°C, respectively, was significantly higher compared to cells incubated at 4°C, but temperature did not significantly affect the phagocytic ability of hemocytes from blue mussel (Figure 7A). In contrast, the phagocytic capacity of none of the aquatic species tested was significantly affected by temperature (Figures 5B,D, 6B,D, 7B,D). The phagocytic index of only the salmon cells incubated at 12°C was significantly higher compared to cells incubated at 4°C (Figure 5C). This temperature effect could not be detected for the phagocytic index of tilapia HK cells (Figure 6C) and blue mussel hemocytes (Figure 7C) although the cells were incubated at higher values, i.e., 12 and 25°C, respectively. The optimized method for phagocytosis assay was well-applied to phagocytic cells from three aquatic animals. The results showed that unlike that of phagocytic cells from fishes, phagocytosis of the cells from mussel was not significantly affected by incubation temperature.

## Discussion

The major advantage of imaging flow cytometry (IFC) over conventional flow cytometry (FC) is its ability to distinguish between false-positive and false-negative events by considering additional features of the captured cellular images (2). The two systems share the basic principle (19). Although IFC has been widely adopted to study mammalian cell types, it is not yet commonly employed to investigate other organisms, including aquatic animals. There is a paucity of appropriate tools such as cell-specific markers, which hampers the wider adoption of new technologies like IFC. Furthermore, the associated protocols require thorough refining before IFC can be used to study cell types from aquatic animals. For example, as the weak and small fluorescence cannot be detected by the system, we employ masking and features within IDEAS software to accurately select the area of interest during image analysis (20). Our study describes procedures to accurately identify cellular phenotypes and quantify phagocytosis by cells from three very different aquatic animals.

In the present IFC study, we could successfully exclude dead cells and cell aggregates and could identify single leukocytes from Atlantic salmon HK based on bright field (BF) area and SSC intensity. We observed two distinct populations: cells located in the low BF area and low SSC intensity, and cells located in the high BF area and high SSC intensity. Our IFC results are in agreement with conventional FC data on HK leukocytes from salmon (21). A study on goldfish primary kidney macrophages also compared IFC results with those from conventional FC data; both systems were used to identify cell sub-populations. Similar dot plots were generated for both flow cytometry systems, indicating that the replacement of forward scatter (FSC) which measures cell size in conventional FC by BF area in IFC (22) is a reliable approach, independent of fish species.

Interestingly, adherent cells from Atlantic salmon HK (R3, macrophage-like cells) were located in a higher BF area than R2 cells from the same organ (Figures 1D,E). The proportion of macrophage-like cells was approximately 45.2%. The macrophage-like cells in the R3 region displayed a similar morphology to that of the adherent TO cells, a cell line originating from salmon HK leukocytes (23). Furthermore, in another study that employed conventional FC, salmon macrophage-like cells were presented in an FSC vs. SSC plot (24). Similar to our gating, the author gated three regions in the plot and assumed that the two higher FSC regions contained macrophage-like cells which was ~56% out of the total number of cells.

After optimizing the method to distinguish between lymphocytes from monocytes/macrophages, magnetic cell sorting (MACS) was performed to sort target lymphocytes using an IgM-specific antibody. The purity of IgM^+^ cells after MACS was 89.8% which is similar to 92% in a salmon study (25). MACS enabled us to ascertain the area of lymphocyte-like cells as defined/interpreted from the BF area vs. SSC intensity plots. The sorted salmon IgM^+^ cells were located in the low BF area and low SSC intensity gate, confirming a close area match to that of the lymphocyte-like cells. Similarly, a previous study on trout HK confirmed lymphocyte localization (low FSC and low SSC) using conventional FC, based on CD4^+^T cell markers (26). In addition, employing conventional FC, percentage of IgM^+^ and IgT^+^ B cells in salmon HK cells were determined by gating the same area (25). The gate areas in Figures 1, 2 confirm the presence of lymphocytes.

After confirming the identity of the B lymphocytes in the low BF area vs. low SSC intensity gate, we explored the phagocytosis of the adherent monocytes/macrophages HK fraction. Phagocytosis is an important initial immune response with final entry of antigens into the phagosomes/lysosomes that stimulates the production of reactive oxygen species (27). Phagocytic activity is influenced by many factors such as cell maturity, cytokine response, antigen presenting cell activation status (28) and the characteristics of phagocytosed antigens or particles (29). We explored the phagocytic activity of salmon HK cells using IFC, which allowed for not only quantification of the number of cells with internalized particles but also the localization of particles inside the cells. The IFC methods for assessing phagocytosis are complex, and researchers are yet to standardize them for different particle types. In the present study, we tested two different types of particles, non-degradable and degradable particles. This is the first IFC study that reports the use of microplastic as non-degradable particles and bio-particles from *E. coli* as degradable particles. Considering the growing debate on microplastic pollution of the marine ecosystem, studying phagocytosis of microplastics by immune cells from aquatic animals can be of particular interest from an environmental perspective. In our studies, with our sensitive IFC methodology, we could clearly detect microplastic particles engulfed by salmon macrophages, although only few particles were detected inside these cells. We, therefore, assume that these cell types can phagocytose microplastics as (foreign) particles. It should be pointed out that the salmon HK phagocytic cells were not able to uptake more microplastics; the reason could be that the cells can efficiently recognize microbe-derived particles (bio particle from *E. coli*) due to their natural antigenicity and phagocytose it more easily than an “unknown particle” such as the microplastic. Furthermore, the microplastic beads are not coated with any compound recognizable by the phagocytic cells, and they are larger compared to the bioparticles.

Compared to microplastic particles, the bio-particles are known to emit fluorescence within cells. However, this occurs only upon acidification, i.e., they emit fluorescence of a particular wavelength, depending on the pH level that the particle encounters. Hence, we suggest the use of fluorescent intensity feature rather than spot count feature to accurately assess the counts of degradable particles in phagocytes. Although a different feature was used to count the number of particles per phagocytic cell, a publication (12) has reported an IFC method for counting internalized fluorescent-labeled bacteria. The author succeeded in distinguishing between cells with high bright detail similarity score and those with low bright detail similarity score; the former one had internalized particles while other cells had external particles. Although the method of Smirnov et al. (12) gives information on the overall degree of phagocytosis in phagocytic cells, it cannot accurately count the internalized particles. Thus, the bright detail similarity score and fluorescent intensity feature are effective in detecting and counting (as in this report) the mean number of internalized particles per phagocytic cell.

Although we did not perform a direct comparison between IFC and conventional FC, from our results we understand that false events such as auto-fluorescence and aggregated particles can be misinterpreted in the case of conventional FC. Pixel and intensity features were adjusted carefully in the present study to exclude the false-positive events. Caution should be exercised when gating phagocytic cells using these features because in IFC, cell size is measured based on pixels, and the sensitivity of the measurement is dependent on the cell size (19). Thus, in order to include the region of interest for analysis, the mask that identifies the intracellular compartment has to be adjusted for different types of cells and particles.

After standardizing the protocols for monocytes/macrophage phagocytosis, we optimized the methods for measuring phagocytosis, using degradable bio-particles, by cells from three very different aquatic animals—two fishes, Atlantic salmon and Nile tilapia and a mollusc, blue mussel—to evaluate the effect of incubation temperature on their phagocytic abilities and capacities. Our results indicated that phagocytosis of cells from the fishes can be affected by the incubation temperature. Although not directly comparable, phagocytosis of human leukocytes was reduced at higher and lower temperature compared to the normal host temperature range (30). Interestingly, the phagocytosis by hemocytes from blue mussel, a eurythermal species that can tolerate a broad temperature range from −1 to 20°C (31, 32), was not affected by incubation temperature.

In summary, IFC was used to study phagocytosis in fish and mussel cells. We were able to identify cell populations and determine the phagocytosis of different kinds of particles by quantifying the number of internalized particles and detecting the localization of particles in the phagocytes. This study provides important information about how IFC can be used in the field of fish immunology and ecotoxicology. Furthermore, the procedures described in this report may have wider application in aquatic sciences, to unravel the effects of microplastic-ingestion by living organisms in the oceans.

## Data Availability Statement

All datasets generated for this study are included in the article/supplementary material.

## Ethics Statement

The animal study was reviewed and approved by National Animal Research Authority in Norway (Mattilsynet).

## Author Contributions

YP and VK conceived and designed the study. YP and IA-G performed the experiments. YP analyzed the data and wrote the first draft of the manuscript while IA-G wrote a section of it. ST, GW and DB provided suggestions to improve the IFC protocols. YP, IA-G, ST, GW, DB, PO, and VK read, revised, and approved the manuscript for submission.

### Conflict of Interest

ST is an employee of Luminex B.V., which is a subsidiary of Luminex Corporation. Luminex Corporation is the manufacturer of the ImageStream^®X^ Mk II Imaging Flow Cytometer. The remaining authors declare that the research was conducted in the absence of any commercial or financial relationships that could be construed as a potential conflict of interest.
